# An Intriguing Case Report of Type 2 Autoimmune Polyendocrine Syndrome Post-SARS-CoV-2: Cause or Coincidence?

**DOI:** 10.2174/0118715303407830250807114958

**Published:** 2025-08-18

**Authors:** Giacomo Voltan, Andrea Graziani, Marianna Torchio, Caterina Mian, Corrado Betterle, Chiara Sabbadin

**Affiliations:** 1 Unit of Endocrinology, University Hospital of Padua, Padua, 35128, Italy;; 2 Department of Medicine (DIMED), University of Padua, Padua, 35128, Italy

**Keywords:** Addison's disease, COVID-19, SARS-CoV-2, adrenal insufficiency, autoimmune polyendocrine syndrome, case report

## Abstract

**Introduction:**

SARS-CoV-2, the virus responsible for COVID-19, is primarily associated with respiratory illness but can also affect multiple organ systems, including the endocrine system. Viral entry into endocrine tissues may lead to immune activation and trigger or unmask autoimmune conditions in individuals who are genetically predisposed. Autoimmune Polyendocrine Syndrome type 2 (APS-2), a rare disorder characterized by autoimmune Addison’s disease (AAD) and autoimmune thyroid disease (AITD), may represent one such manifestation.

**Case Presentation:**

We report the case of a 36-year-old male who developed APS-2 following a mild SARS-CoV-2 infection. Two months post-infection, the patient experienced asthenia, hypotension, gastrointestinal symptoms, and weight loss. Laboratory investigations revealed undetectable morning cortisol, positive 21-hydroxylase and thyroid-peroxidase autoantibodies, elevated ACTH and renin, and subclinical hypothyroidism—consistent with a diagnosis of APS-2 (AAD and Hashimoto’s thyroiditis). Treatment with cortisone acetate and fludrocortisone led to clinical improvement. No previous history of autoimmune disease was reported. A review of the literature identified only four similar case reports, with varying timelines between SARS-CoV-2 infection and APS-2 diagnosis, suggesting that the infection may act as a trigger in predisposed individuals.

**Conclusion:**

This case adds to limited evidence suggesting a possible link between SARS-CoV-2 infection and the onset or unmasking of APS-2. While a direct causal role of the virus remains uncertain, SARS-CoV-2 may function as an environmental trigger, accelerating the transition from subclinical to clinical autoimmunity in genetically susceptible patients. This observation supports the need for clinical vigilance in post-COVID-19 patients presenting with nonspecific but suggestive endocrine symptoms.

## 1 INTRODUCTION

SARS-CoV-2, the virus responsible for COVID-19 and the recent global pandemic, has caused approximately 630 million cases worldwide [1, 2], with over 6.6 million deaths [3], primarily due to the rapid spread of infection and severe viral pneumonia leading to acute respiratory distress as the most serious outcome [4]. However, it might lead to other systemic manifestations including cardiovascular, neurological, gastrointestinal, and endocrine complications [5, 6].

Indeed, viral entry into cells has been observed in several endocrine glands, including the thyroid, testes, ovaries, adrenal glands, pituitary gland, and endocrine pancreas [7-10]. For example, in the thyroid, SARS-CoV-2 may trigger a “cytokine storm” and hyperactivation of the immune response, potentially leading to subacute thyroiditis or autoimmune thyroid diseases (AITDs), such as Graves’ disease (GD) or Hashimoto’s thyroiditis (HT), particularly in genetically susceptible individuals [9, 10].

These mechanisms have also been described as possible direct or indirect causes of primary adrenal insufficiency (PAI) during or after SARS-CoV-2 infection [11, 12].

In a previous study, SARS-CoV-mediated damage to pancreatic β-cells was proposed as a mechanism behind the development of ‘acute diabetes’ in patients with SARS [13]. Although coronavirus is not included in the list of viruses implicated in the etiopathogenesis of type 1 diabetes mellitus (T1DM), SARS-CoV (and perhaps SARS-CoV-2) could be a potential trigger for its development [8].

The pathogenetic mechanisms of autoimmune endocrine diseases associated with SARS-CoV-2 are still poorly understood but may involve molecular mimicry, bystander activation, and loss of immune tolerance [14].

Here, we present the case of an adult patient who was diagnosed with type 2 Autoimmune Polyendocrine Syndrome (APS-2), characterized by Autoimmune Addison’s disease (AAD) and Hashimoto’s thyroiditis (HT), following SARS- CoV-2 infection. We performed a review of the literature regarding the possible association between APS-2 and SARS- CoV-2.

We strictly adhered to the CARE guidelines during the writing of this manuscript.

## 2 CASE REPORT

In July 2022, a 36-year-old man contracted a mild form of COVID-19. Two months later, he began experiencing worsening asthenia, dizziness, gastrointestinal issues, and unintentional weight loss. His family and personal history were negative for chronic or autoimmune diseases. In October, his general practitioner recommended biochemical tests that revealed low TSH levels, positive thyroid peroxidase autoantibodies (TPO-Abs), the absence of IgA anti-transglutaminase autoantibodies, undetectable morning serum cortisol levels, and normal sodium and potassium levels. Suspecting adrenal insufficiency, the patient was referred to our Endocrine Unit. Physical examination revealed hypotension (95/60 mmHg) and mucocutaneous hyperpigmentation. Considering the biochemical and clinical findings, we started drug therapy with cortisone acetate 25 mg divided into two daily doses.

After two months, the patient reported improvement in symptoms, weight, and blood pressure. Measurement of 21-hydroxylase autoantibodies (21OH-Abs) was performed and returned positive. Blood tests revealed normal sodium and potassium levels, elevated renin and ACTH, and low serum aldosterone levels. Additionally, subclinical hypothyroidism was observed, with the absence of other autoantibodies, such as those against parietal cells and the endocrine pancreas.

Thus, the diagnosis of APS-2 (AAD and HT) was confirmed, and fludrocortisone therapy was added to the cortisone acetate regimen.

Blood test results and clinical features are reported in Table **1**.

## 3 REVIEW OF THE LITERATURE

We conducted a literature review on the current understanding of the potential association between APS-2 and SARS-CoV-2 infection. To identify reported case reports of “APS and COVID-19,” we queried the PubMed database using the following keywords, combined in various ways: “Addison” or “Addison’s disease” or “Autoimmune adrenal insufficiency” AND “COVID-19” or “SARS-CoV-2.” We excluded non-English articles, reviews, and reports concerning adrenal crisis in patients with pre-existing Addison’s disease, as well as PAI following SARS-CoV-2 infection without evaluation of anti-21OH antibodies. This search yielded only four relevant case reports [15-18].

Bhattarai *et al.* [15] described a 19-year-old female who developed generalized malaise, nausea, multiple episodes of emesis, and decreased oral intake four days after the discovery of a positive SARS-CoV-2 test. Upon hospital admission, she exhibited an altered mental state and posturing, prompting an emergency room (ER) visit. She was intubated and treated with isotonic fluids and a norepinephrine infusion for persistent hypotension. Laboratory findings demonstrated Na 110 mmol/L, K 6.8 mmol/L, TSH 22 mIU/L, and low cortisol levels; therefore, an intravenous dose of 100 mg of hydrocortisone was started.

The autoimmune screening revealed the presence of 21OH-Abs and thyroglobulin autoantibodies (Tg-Abs), consistent with an APS-2 (AAD and HT). Along with neurological status improvement, the patient reported that salt craving, fatigue, and hyperpigmentation of her buccal mucosa had started 6 months before COVID-19. These aspects suggest that AAD was already present before SARS-CoV-2 infection. Indeed, hyperpigmentation is not an acute sign, appearing months after the increase of ACTH, as demonstrated in patients positive for 21OH-Abs progressing to clinical AAD [19].

Therefore, this case of APS-2 could not be considered a consequence of SARS-CoV-2 infection.

Sánchez [16] reported the case of a 64-year-old woman with a personal history of type 2 DM and HT. Five months after an asymptomatic SARS-CoV-2 infection, she experienced fatigue and unintentional weight loss. She visited the ER twice with nausea, vomiting, poor oral intake, and low sodium levels, initially treated with fluid restriction, suspecting syndrome of inappropriate antidiuresis. Returning with worsening abdominal pain and new oral mucosa hyperpigmentation, she had low blood pressure (93/66 mmHg) and severe hyponatremia, which resolved with intravenous hydrocortisone. ACTH levels were very high, and 21OH-Abs were present, consistent with AAD. Even this case was affected by APS-2. Considering that the patient reported hyperpigmentation five months after SARS-CoV-2 infection, it is possible that the infective disease uncovered a potential or subclinical AAD.

Flokas [17] described a 14-year-old female with a history of vitiligo diagnosed at the age of 9. She arrived at the ER in shock after a week of fever, lethargy, and gastrointestinal issues. Three weeks earlier, she had been exposed to SARS- CoV-2 at home. Laboratory tests showed hyponatremia (129 mmol/L) and a positive SARS-CoV-2 PCR. She was resuscitated with normal saline and started on an epinephrine drip. Further tests revealed undetectable cortisol, elevated ACTH, positive 21OH-Abs, and normal adrenal imaging, confirming AAD. Thyroid status assessment highlighted severe hypothyroidism (TSH 131 mIU/L) with positive thyroid autoantibodies.

Intravenous hydrocortisone was initiated, improving her clinical condition. In conclusion, even in this case, APS-2 (vitiligo, AAD, and HT) may have been triggered by SARS- CoV-2 infection in a susceptible patient with an underlying autoimmune condition.

Sambangi [18] described a 15-year-old female admitted to the ER for a Multisystem Inflammatory Syndrome in Children due to SARS-CoV-2 infection. She was treated with intravenous immunoglobulin, low-dose aspirin, and methylprednisolone, which was continued for nine months after discharge. Three months after stopping the steroids, she returned to the hospital due to weight loss, difficulty breathing, polyuria, polydipsia, nausea, vomiting, and fatigue. Exams confirmed new-onset T1DM with positive anti-Glutamic Acid Decarboxylase autoantibodies, and insulin treatment was initiated. Subsequently, she developed COVID-19 for a second time and returned to the ER after two months for persistent abdominal pain, dizziness, and nausea.

On examination, the patient had episodes of hypotension and arrhythmia. Laboratory findings demonstrated a PAI with 21OH-Abs positivity and a thyrotoxicosis due to GD. Even this case was consistent with an APS-2 (T1DM, AAD, and GD). In children and adolescents, APS-2 usually manifests initially with T1DM, followed by AAD with or without AITD. On the contrary, in adults it usually starts with AITD, followed by AAD and rarely by T1DM [20].

In this case, the link between infection and T1DM, which appeared a year after the first SARS-CoV-2 infection and prolonged steroid treatment, is unclear. As the Latins said, 'post hoc' does not always mean 'propter hoc.' Moreover, AAD and GD manifested simultaneously after the second SARS-CoV-2 infection, potentially uncovering pre-existing autoimmune diseases.

## 4 DISCUSSION

APS-2 is a rare syndrome, characterized by AAD associated with AITD and/or T1DM [21, 22]. It is a chronic disease characterized by three distinct phases (potential, subclinical, and clinical), marked by the presence of circulating autoantibodies [23, 24].

During the natural history of the syndrome, some symptoms or signs may be present before clinical diagnosis. Specifically, regarding AAD, mucocutaneous hyperpigmentation is a sign of prolonged elevation of ACTH [19]. Additionally, the autoimmune diseases included in APS-2 are multifactorial, influenced by genetic predisposition, epigenetic factors, and endogenous and environmental elements, such as toxic chemicals, dietary components, gut dysbiosis, and infections [24-27]. Epidemiological data suggest infections may play a role in the development of these autoimmune diseases; however, their long natural course makes it difficult to pinpoint any specific virus contributing to the disease’s initial stages [28].

Several immunological mechanisms may underlie the association between SARS-CoV-2 infection and the subsequent onset or unmasking of autoimmune endocrine diseases, although a direct pathogenic role of the virus remains uncertain [29, 30].

One of the most studied mechanisms is molecular mimicry, in which viral antigens share structural homology with host self-antigens. This similarity can trigger the activation of autoreactive T and B cells that mistakenly target endocrine tissues [31-33].

Another plausible mechanism is bystander activation, where infection-induced inflammation causes the release of cytokines and danger-associated molecular patterns that stimulate antigen-presenting cells. This proinflammatory environment may non-specifically activate previously anergic or suppressed autoreactive T cells, thereby promoting autoimmunity independently of specific viral epitopes [34].

Cytokine-mediated tissue damage is also a significant contributor. SARS-CoV-2 infection is frequently associated with elevated levels of proinflammatory cytokines, particularly interleukin-6, tumor necrosis factor-alpha, and interferon-gamma [35]. These cytokines not only sustain systemic inflammation but also promote increased expression of MHC class I and II molecules on endocrine cells, enhancing autoantigen presentation. In this context, autoreactive T cells may become activated and initiate an immune attack against previously tolerated self-antigens. This process is further facilitated by epitope spreading, where the initial immune response targeting a limited number of epitopes broadens to include additional epitopes on the same or different autoantigens [33, 36].

Finally, the disruption of immune tolerance through SARS-CoV-2–induced lymphopenia and alteration of regulatory T cell (Treg) function, has also been proposed as a contributing factor. The transient reduction in Tregs and natural killer cells observed in COVID-19 may further impair peripheral tolerance mechanisms, potentially allowing autoimmunity to emerge [37]. Although these mechanisms are biologically plausible and supported by parallels in other post-viral autoimmune diseases, direct causal evidence remains limited. It is more likely that SARS-CoV-2 acts as an environmental trigger of an already ongoing subclinical autoimmune process in genetically susceptible individuals, as may have occurred in the present case.

The timeline between SARS-CoV-2 infection and the clinical onset of endocrine autoimmune diseases is fundamental. We have discussed how these diseases may have been present before or manifested during or after SARS- CoV-2 infection. However, to consider a virus pathogenic, it should be identified during seroconversion, which often occurs months or years before clinical manifestations. If clinical symptoms align with the timing of viral infection, the virus may act only as a trigger for a latent autoimmune disease. Alternatively, SARS-CoV-2 can have an acute direct effect on endocrine target organs, as demonstrated by several cases of clinical non-autoimmune diseases occurring simultaneously, such as subacute thyroiditis, PAI due to adrenal hemorrhage, infarction, or vascular diseases, or acute T1DM without the presence of organ-specific autoantibodies [7-11].

Furthermore, SARS-CoV-2 infection is known to cause significant disruption in the balance between innate and adaptive immunity, often reflected by altered hematological indices, such as the neutrophil-to-lymphocyte ratio (NLR), monocyte-to-lymphocyte ratio (MLR), and elevated C-reactive protein levels. These markers have been shown to correlate with disease severity and prognosis, particularly in cases involving respiratory complications [38, 39]. In our patient, the only available data was the blood count at the initial evaluation, which showed no elevation of NLR or MLR, possibly due to the mild form of COVID-19 experienced.

## CONCLUSION

SARS-CoV-2 infection has been linked to several clinical manifestations, including endocrine diseases. Among these, autoimmune thyroid disease and PAI have been reported. The underlying pathogenetic mechanisms of PAI remain poorly understood; however, possible pathways may involve molecular mimicry, bystander activation, and loss of immune tolerance. As discussed in our clinical case, it is unlikely that the development of APS-2 during or after SARS- CoV-2 infection is directly caused by the virus. Rather, the virus may have uncovered an autoimmune syndrome that was already present in a subclinical form. This perspective can also be applied to the association between other viral infections and the subsequent onset of autoimmune diseases [40]. It is crucial to consider this possibility, given the potentially severe and even fatal consequences of undiagnosed PAI.

## Figures and Tables

**Table 1:** Patient’s physical biochemical exams in October 2022 and in January 2023.

**-**	**Reference Range**	**First Evaluation**	**Two Months Later**
Blood pressure (mmHg)	120/80	95/60	120/80
White cells count (10^9/L)	4.4-11.00	5.25	n.a.
Lymphocytes count (10^9/L)	1.1-4.8	2.71	n.a.
Neutrophils count (10^9/L)	1.8-7.8	1.59	n.a.
Monocytes count (10^9/L)	0.2-0.96	0.8	n.a.
NLR	n.a.	0.59	n.a.
MLR	n.a.	0.30	n.a.
TSH (mIU/L)	0.4-4	0.06	6.02
Na (mmol/L)	135-145	138	139
K (mmol/L)	3.5-5	4.9	4.6
8 am Cortisol (mcg/dL)	5-18	< 0.1	10
Anti-TG Ab	Positive/Negative	Positive	Not repeated
Anti-TPO Ab	Positive/Negative	Positive	Not repeated
ACTH (pg/mL)	10-60	n.a.	1250
Anti-21-hydroxylase Ab	Positive/Negative	n.a.	Positive
Renin (UI/mL)	3-39	n.a.	226
Aldosterone (pg/mL)	25-250	n.a.	10.9

**Abbreviations:** Ab: antibody; ACTH; adrenocorticotropic hormone; TSH: thyroid stimulating hormone; TG: thyroglobulin; TPO: thyroid-peroxidase; NLR: neutrophils-to-lymphocytes ratio; MLR monocytes-to-lymphocytes ratio; na: not available.

## Data Availability

All data generated or analysed during this study are included in this published article.

## LIST OF ABBREVIATIONS

APS-2: Autoimmune Polyendocrine Syndrome type 2
AAD: Autoimmune Addison’s Disease
ATD: Autoimmune Thyroid Disease
GD: Graves’ Disease
HT: Hashimoto’s Thyroiditis
PAI: Primary Adrenal Insufficiency
ER: Emergency Room
Tg-Abs: Thyroglobulin Autoantibodies
MLR: Monocyte-to-lymphocyte Ratio
NLR: Neutrophil-to-lymphocyte Ratio

## STANDARDS OF REPORTING

CARE guidelines were followed.
